# Exercise hemodynamics in adults with Fontan circulation assessed by noninvasive impedance cardiography

**DOI:** 10.3389/fcvm.2026.1876375

**Published:** 2026-09-04

**Authors:** Agnieszka Bartczak-Rutkowska, Ewa Straburzyńska-Migaj, Sonia Nartowicz, Magdalena Dudek, Aleksandra Ciepłucha, Agata Markiewicz, Maciej Lesiak, Olga Trojnarska

**Affiliations:** Department of Cardiology, Poznan University of Medical Sciences, Poznan, Poland

**Keywords:** congenital heart defect, exercise capacity, Fontan circulation, noninvasive impedance cardiography, PhysioFlow

## Abstract

**Background:**

Exercise intolerance is a hallmark of Fontan physiology despite preserved resting hemodynamics. The relative roles of stroke volume, chronotropic response, and vascular adaptation during exercise remain incompletely defined in adults with Fontan circulation.

**Methods:**

In this prospective observational study, 27 adults with Fontan circulation were enrolled, of whom 22 with complete cardiopulmonary exercise testing (CPET) and impedance cardiography data were included in the final analysis. Twenty-five healthy controls underwent the same protocol. Stroke volume (SV), heart rate (HR), cardiac output (CO), cardiac index (CI), and systemic vascular resistance (SVR) were assessed at rest, at the ventilatory threshold (VT), and at peak exercise. Between-group differences and within-Fontan exploratory correlations between hemodynamic parameters and CPET-derived indices were analyzed.

**Results:**

Resting hemodynamics were comparable between groups. During exercise, Fontan patients exhibited a markedly attenuated cardiovascular response, with lower peak HR (*p* < 0.001), CO (*p* < 0.001), and CI (*p* < 0.001), and higher SVR (*p* = 0.01). Stroke volume was reduced at peak exercise (*p* = 0.045) but did not correlate with exercise capacity. In contrast, peak HR correlated with VO_2_% predicted (*r* = 0.52, *p* = 0.01), and chronotropic incompetence was associated with reduced cardiac index (*r* = −0.44, *p* = 0.043). SVR correlated inversely with VO_2_ (*r* = −0.47, *p* = 0.03).

**Conclusions:**

Adults with Fontan circulation demonstrate preserved resting hemodynamics but impaired cardiovascular reserve during exercise. Our findings suggest that impaired chronotropic response was closely associated with reduced exercise reserve, but should be interpreted as part of preload-limited Fontan physiology rather than as an isolated causal mechanism. Altered vascular response may contribute, although SVR estimates are relative because CVP was not directly measured. Noninvasive exercise hemodynamic monitoring provides complementary mechanistic insight beyond CPET alone.

## Introduction

Improved survival of patients with single-ventricle physiology after the Fontan procedure has led to a growing population of these adult patients (1). Despite favorable long-term survival outcomes, many patients experience a progressive decline in exercise tolerance, reduced quality of life, and an increased risk of late complications such as heart failure, arrhythmias, or multiorgan dysfunction (2, 3). The Fontan circulation represents a unique physiological model in which the absence of a subpulmonary ventricle means that blood flow through the pulmonary circulation depends on passive venous return and low pulmonary vascular resistance (2, 4).

An important clinical issue is that many patients with Fontan circulation present with normal or only mildly impaired resting hemodynamic parameters and remain in a low New York Heart Association (NYHA) functional class (I–II), despite a clearly reduced exercise capacity (5). The discrepancy between subjective assessment of exercise tolerance and objective measures of physical performance suggests that resting evaluation may not reflect the true circulatory reserve, and that dynamic assessment during exercise is crucial for identifying significant physiological abnormalities (6, 7).

Noninvasive impedance cardiography enables continuous, beat-to-beat assessment of central hemodynamic parameters during physical exercise (8). When combined with exercise testing, this method allows for a detailed analysis of changes in stroke volume, heart rate, cardiac output, and vascular resistance beyond standard cardiopulmonary exercise testing (CPET) parameters and resting imaging studies (8, 9). Furthermore, considering the inherent risks and limited feasibility associated with direct invasive measurements of stroke volume and cardiac output during exercise, noninvasive exercise assessment using impedance cardiography validated against the direct Fick method provides a practical and safer alternative for routine clinical practice (8). However, validation data for impedance cardiography come primarily from selected non-Fontan populations. Therefore, impedance-derived values in Fontan physiology should be interpreted mainly as relative exercise responses rather than definitive invasive hemodynamic measurements. Noninvasive impedance cardiography has been successfully applied not only in patients with heart failure but also in those with congenital heart defects (10, 11). To the best of our knowledge, only two studies have evaluated exercise hemodynamic profiles in Fontan patients using impedance cardiography, both mainly in younger cohorts (12, 13).

The aim of our study was a comprehensive assessment of central and peripheral hemodynamic parameters during physical exercise in adult patients with Fontan circulation using noninvasive impedance cardiography combined with cardiopulmonary exercise testing.

## Materials and methods

The study included 27 adult patients with Fontan circulation who were followed at a tertiary congenital heart disease center. The mean age of the Fontan cohort was 30.7 ± 7.0 years, and 37% (*n* = 10) were male. The Fontan procedure had been performed at a mean age of 8.0 ± 3.7 years, with a mean postoperative duration of 22.6 ± 6.5 years. Underlying congenital heart defects included tricuspid atresia (37%), double inlet left ventricle (22.2%), double outlet right ventricle (18.5%), pulmonary atresia (11.1%), mitral atresia (3.7%), hypoplastic left heart syndrome (3.7%), and congenitally corrected transposition of the great arteries (3.7%). Most patients had undergone total cavopulmonary connection (TCPC) (92.6%), while 7.4% had an atriopulmonary connection. Fenestration was present in 22.2% of patients. At the time of evaluation, 63% of patients were classified as NYHA class I and 37% as NYHA class II. Dominant ventricular morphology was left ventricular in 70.4% and right ventricular in 29.6% of patients. Five patients were unable to undergo both cardiopulmonary exercise testing and simultaneous impedance cardiography because of non-cardiovascular limitations. Therefore, the final analyses included 22 Fontan patients with complete CPET and impedance cardiography datasets.

A control group of 25 healthy subjects, matched for age, sex, and body mass, without known cardiovascular disease was included for comparison of exercise hemodynamic responses.

### Study design

This was a prospective observational study assessing resting and exercise-induced cardiovascular responses using noninvasive impedance cardiography. All participants underwent incremental exercise testing with continuous hemodynamic monitoring. Hemodynamic parameters were compared between Fontan patients and healthy controls at predefined exercise stages. Hemodynamic parameters were evaluated at predefined exercise stages. Because the study was observational and exploratory, associations between exercise hemodynamics and CPET-derived variables were interpreted as hypothesis-generating.

### Hemodynamic assessment

Hemodynamic parameters were assessed noninvasively using transthoracic impedance cardiography (PF07 Enduro PhysioFlow, Petit Ebersviller, France). This technique estimates beat-to-beat changes in stroke volume based on the analysis of thoracic impedance signal morphology and its first derivative during the cardiac cycle, reflecting variations in blood volume and flow velocity in the ascending aorta. The following parameters were recorded: stroke volume (SV, mL), heart rate (HR, beats/min), cardiac output (CO, L/min), cardiac index (CI, L/min/m^2^), systemic vascular resistance (SVR, dyn·s/cm^5^). SV was calculated by the proprietary PhysioFlow algorithm using the maximal rate of impedance change (dZ/dt_max), systolic impedance amplitude, ventricular ejection time, and correction factors incorporating heart rate and arterial blood pressure. CO was derived as the product of SV and HR, and CI was calculated by indexing cardiac output to body surface area. SV was analyzed at three predefined stages of the exercise protocol: SV1, at rest after achieving stable hemodynamic conditions; SV2, was assessed at the ventilatory threshold (VT), determined using the V-slope method and confirmed by conventional ventilatory criteria; and SV3, at peak exercise, defined as the highest workload achieved immediately before test termination. Beat-to-beat values were averaged over consecutive 5-second intervals with impedance signal quality ≥95% by the PhysioFlow software before further analysis. The ventilatory threshold was chosen to represent a physiologically meaningful submaximal exercise level, allowing assessment of stroke volume reserve under standardized conditions independent of maximal exercise performance. SVR was calculated indirectly from impedance-derived cardiac output and noninvasively measured arterial pressure. As central venous pressure was not directly measured, a fixed CVP assumption was applied uniformly in the calculation of SVR. Because CVP is characteristically elevated and exercise-responsive in Fontan circulation, SVR values should be interpreted as relative estimates rather than absolute measures of systemic vascular tone.

### Cardiopulmonary exercise protocol

Cardiopulmonary exercise testing (CPET) was performed on a treadmill using the Vyntus CPX system (Jaeger, Germany) with a Ramp protocol. Patients were encouraged to exercise to volitional exhaustion, and respiratory gas exchange was measured breath-by-breath. Gas analyzers were calibrated before each test using standard reference gases. Exercise parameters were analyzed as 10-second averages and included oxygen uptake (VO_2_ and VO_2_%), resting and peak heart rate (HRrest, HRpeak), heart rate reserve (HRR). HRR was calculated as (HRpeak − HRrest)/(HRmax − HRrest), where HRmax denotes the age-predicted maximal heart rate calculated as 220 minus age. Chronotropic incompetence was defined as HRR ≤0.80, or ≤0.62 in patients receiving beta-blocker therapy. Additional parameters included percent-predicted heart rate (HR%); oxygen pulse (VO_2_/HR), ventilatory efficiency (VE/VCO_2_ slope), and respiratory exchange ratio (RER). Peak VO_2_ and related indices were defined as the highest 30-second averaged VO_2_ achieved during exercise. Importantly, peak VO_2_ rather than VO_2_max was reported, in line with current practice in Fontan patients. Peak exercise values were available for all 22 patients included in the final study cohort. All completed CPETs were included regardless of whether an RER ≥1.10 was achieved, as reaching an RER ≥1.10 is often not feasible in patients with Fontan circulation.

The Bioethical Committee of Poznan University of Medical Sciences approved the protocol—Board Review 475/19. Each participant provided informed consent to participate in this study.

### Statistical analysis

Continuous variables are presented as mean (standard deviation) or median (min–max range), depending on data distribution, as assessed by the Shapiro–Wilk test. Categorical variables are presented as counts and percentages. Between-group comparisons of categorical variables were performed using Fisher's exact test. Between-group comparisons at each exercise stage were performed using the unpaired Student's *t*-test for normally distributed variables or the Mann–Whitney *U* test for non-normally distributed variables. Given the exploratory nature of the study, no adjustment for multiple comparisons was applied. Correlation analyses between peak exercise hemodynamic parameters (Stage 3) and CPET-derived variables were performed exclusively within the Fontan cohort using Pearson's correlation coefficient for normally distributed data or Spearman's rank correlation coefficient for non-normally distributed data. A *p*-value <0.05 was considered statistically significant.

## Results

### Baseline characteristics

Baseline characteristics of all enrolled Fontan patients (*n* = 27) and controls (*n* = 25) are presented in Table 1. Five Fontan patients were unable to complete both CPET and impedance cardiography; therefore, all subsequent hemodynamic, CPET analyses and correlation analyses were performed in the remaining 22 patients with complete datasets. The groups were comparable with respect to sex distribution, age, weight, BMI, and BSA, with no significant differences observed. Additionally, NT-proBNP levels were significantly higher in the Fontan group compared to controls (*p* < 0.001).

**Table 1:** Baseline characteristics of all enrolled Fontan patients and the control group.

Parameter	Fontan (*n* = 27)	Controls (*n* = 25)	*p*
Male (%)	10 (37%)	10 (40%)	0.92
Age (years)	30.7 ± 7	32.6 ± 5.6	0.45
Weight (kg)	65 ± 11	71.8 ± 14	0.13
BMI (kg/m^2^)	22.9 ± 3.1	23.6 ± 3.6	0.54
BSA (m^2^)	1.7 (1.5–2.2)	1.9 (1.6–2.2)	0.14
NTproBNP	241 (46–905)	52.5 (17–120)	**<0** **.** **001**

BMI, body mass index; BSA, body surface area.

Bold values indicate statistical significance (*p* < 0.05).

### Hemodynamic parameters at rest (Stage 1)

Among the 22 Fontan patients included in the final analysis, all PhysioFlow and CPET measurements were available and suitable for analysis. At rest, Fontan patients and healthy controls demonstrated comparable central hemodynamic parameters (Table 2). Stroke volume did not differ between groups (*p* = 0.5). Resting HR was similar (*p* = 0.6), as were CO (*p* = 0.8), CI (*p* = 0.7) and SVR (*p* = 0.9).

**Table 2:** Comparison of hemodynamic (PhysioFlow) and cardiopulmonary exercise parameters at successive stages of exercise between patients with Fontan circulation and the control group.

Parameter	Fontan (*n* = 22)	Controls (*n* = 25)	*p*
Hemodynamic parameters (PhysioFlow) at rest			
SV1 (mL)	56.3 ± 17.9	61.2 ± 23.5	0.5
HR1 (beat/min)	89.3 ± 17.8	86.2 ± 9.8	0.6
CO1 (L/min)	5 ± 1.7	5.2 ± 2	0.8
CI1 (L/min/m^2^)	2.9 ± 1	2.8 ± 1	0.7
SVR1 (dyn.s/cm^5^)	1,520 ± 478	1,545 ± 626	0.9
Hemodynamic parameters (PhysioFlow) at VT threshold			
SV2 (mL)	78.1 ± 16.2	86.4 ± 38.7	0.4
HR2 (beat/min)	110.3 ± 23.5	130.1 ± 19.2	**0** **.** **03**
CO2 (L/min)	8.5 ± 2.1	10.9 ± 4.3	**0**.**047**
CI2 (L/min/m^2^)	5 ± 1.3	5.8 ± 2.1	0.2
SVR2 (dyn.s/cm^5^)	895 ± 170	826 ± 306	0.4
Hemodynamic parameters (PhysioFlow) at peak exercise			
SV3 (mL)	85.9 ± 24.7	112.6 ± 46.8	**0**.**045**
HR3 (beat/min)	135.9 ± 28.3	179.5 ± 12.4	**<0**.**001**
CO3 (L/min)	11.7 ± 3.7	20.2 ± 8.7	**<0**.**001**
CI3 (L/min/m^2^)	6.9 ± 2.1	10.7 ± 3.9	**<0**.**001**
SVR3 (dyn.s/cm^5^)	628 (398–5,295)	456 (266–803)	**0**.**01**
Cardiopulmonary exercise testing parameters (CPET)			
VO_2_ (mL/kg/min)	20.9 ± 5.2	35.5 ± 7.7	**<0**.**001**
VO_2_%	53.3 ± 12	114.9 ± 19.3	**<0**.**001**
Oxygen pulse (mL/beat)	10 ± 2.9	14.8 ± 3.7	**<0**.**001**
HRR	0.5 ± 0.23	0.9 ± 0.11	**<0**.**001**
HR%	77 (52–93)	93.5 (88–110)	**<0**.**001**
VE/VCO_2_ slope	27.8 (19.8–49.4)	22.4 (19–26.5)	**0**.**001**
RER	1 (0.8–1.2)	1.2 (1.1–1.3)	**<0** **.** **001**
Chronotropic incompetence	17 (77.3%) no β-b 9/17 (53%) with β-b 8/17 (47%)	-	

1, rest; 2, submaximal exercise; 3, peak exercise; SV, stroke volume; HR, heart rate; CO, cardiac output; CI, cardiac index; SVR, systemic vascular resistance; VO_2_, oxygen uptake; VO_2_%, percentage of predicted peak oxygen uptake; HRR, heart rate reserve; HR%, predicted heart rate; VE/VCO_2_ slope, ventilation vs. carbon dioxide output slope; RER, respiratory exchange ratio; β-b, beta-blockade.

Bold values indicate statistical significance (*p* < 0.05).

### Hemodynamic response at submaximal exercise (Stage 2)

During submaximal exercise, SV remained similar between Fontan patients and controls (*p* = 0.4). However, HR and CO were significantly lower in the Fontan group (*p* = 0.03; *p* = 0.047). CI did not differ significantly (*p* = 0.2). Median SVR values were comparable between groups (*p* = 0.4).

### Hemodynamic response at peak exercise (Stage 3)

**Figure 1:**
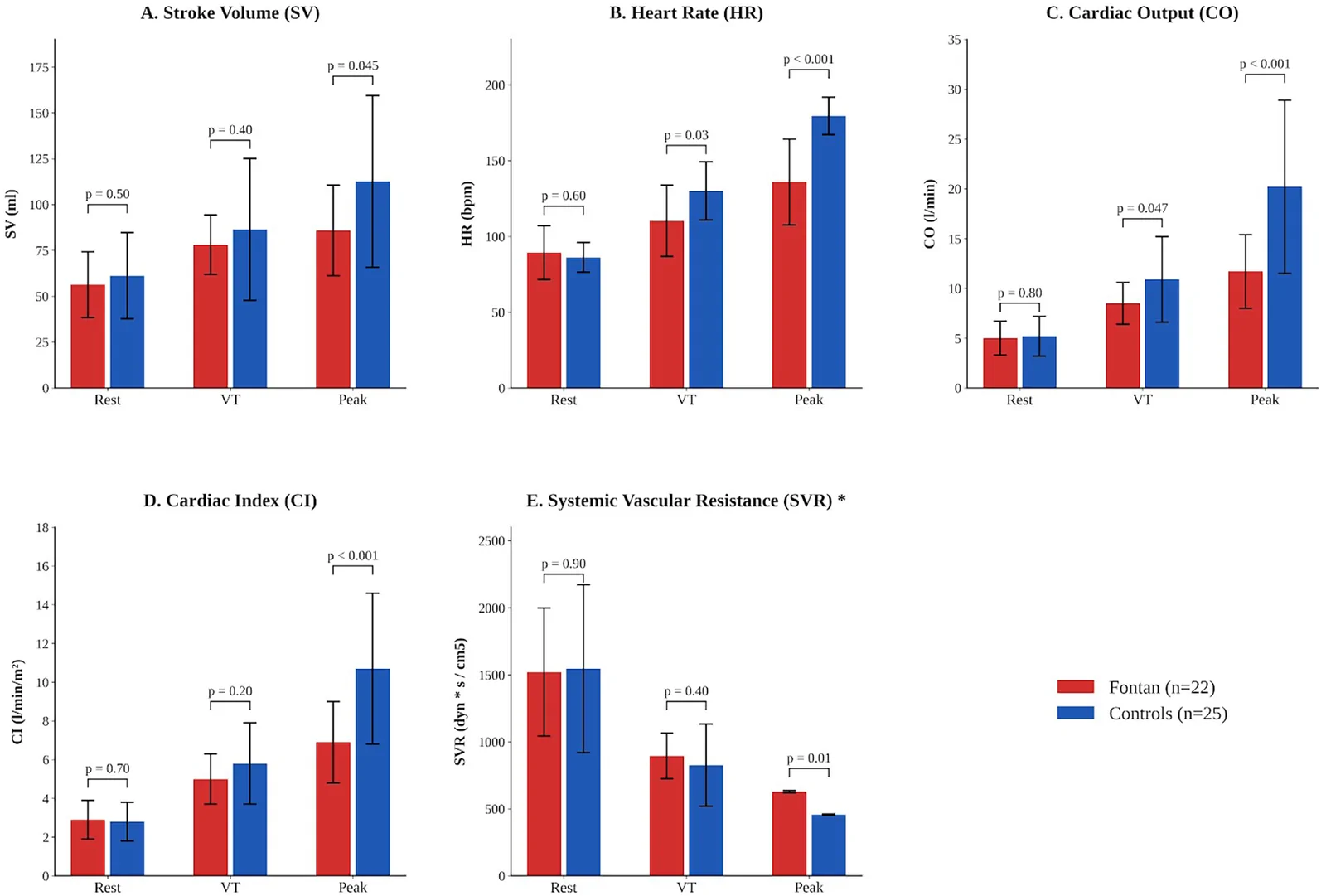
Comparison of hemodynamic parameters at rest, ventilatory threshold (VT), and peak exercise in adults with Fontan circulation (*n* = 22) and healthy controls (*n* = 25). Panels show stroke volume (SV; **A**), heart rate (HR; **B**), cardiac output (CO; **C**), cardiac index (CI; **D**), and systemic vascular resistance (SVR; **E**). * SVR was calculated under the assumption of constant central venous pressure. VT, ventilatory threshold. Bars represent mean ± SD [SVR: median (min–max)]. *P* values indicate between-group comparisons at each exercise stage.

At peak exercise, Fontan patients exhibited an attenuated cardiovascular response compared with controls (Figure 1; Table 2). HR, CO and CI were significantly lower in Fontan patients (all *p* < 0.001). Stroke volume was lower in Fontan patients at peak exercise (*p* = 0.045). SVR was higher in the Fontan group (*p* = 0.01). Among the 22 Fontan patients included in the final analysis, 17 (77.3%) met the criteria for chronotropic incompetence. Of these, 8 (47%) were receiving beta-blocker therapy, including 2 patients with a pacemaker.

### Correlations within the Fontan group between peak exercise hemodynamics and CPET parameters

Correlation analyses at peak exercise (Stage 3) within the Fontan group are presented in Table 3. Stroke volume did not correlate significantly with any CPET-derived variables. In contrast, HRpeak showed a positive correlation with VO_2_% predicted (*r* = 0.52, *p* = 0.01). CO and CI demonstrated positive correlations with VO_2_ (CO: *r* = 0.53; *p* = 0.01; CI: *r* = 0.42; *p* = 0.045) and VO_2_% predicted (CO: *r* = 0.46; *p* = 0.03; CI: *r* = 0.50; *p* = 0.01). Importantly, SVR at peak exercise correlated negatively with RER (*r* = −0.49; *p* = 0.02), VO_2_ (*r* = −0.47, *p* = 0.03) and VO_2_% (*r* = −0.44, *p* = 0.04). Chronotropic incompetence was associated with lower CI (*r* = −0.44, *p* = 0.043), whereas no significant correlations were observed with SV or SVR parameters.

**Table 3:** Correlations between peak exercise hemodynamic parameters (Stage 3) and selected cardiopulmonary exercise testing variables within the Fontan patients with complete CPET and impedance cardiography data (*n* = 22).

Parameter	SV3	HRpeak	CO3	CI3	SVR3
VO_2_	ns	0.39 0.06	0.53 **0.01**	0.42 **0.045**	−0.47 **0.03**
VO_2_%	ns	0.52 **0.01**	0.46 **0.03**	0.5 **0.01**	−0.44 **0.04**
Oxygen pulse	ns	ns	ns	ns	ns
VE/VCO_2_	ns	ns	ns	ns	ns
Chronotropic incompetence	ns	- -	−0.33 0.13	−0.44 **0.043**	0.16 0.47
RER	ns	ns	0.38 0.08	0.4 0.06	−0.49 **0.02**
NYHA	0.45 **0.03**	−0.45 **0.03**	ns	ns	ns

SV, stroke volume; HRpeak, heart rate at peak; CO, cardiac output; CI, cardiac index; SVR, systemic vascular resistance; VO_2_, oxygen uptake; VO_2_%, percentage of predicted peak oxygen uptake; VE/VCO_2_ slope, ventilation vs. carbon dioxide output slope; RER, respiratory exchange ratio; NYHA, New York Heart Association functional class.

Bold values indicate statistical significance (*p* < 0.05).

NYHA functional class correlated positively with SV (*r* = 0.45; *p* = 0.03) and negatively with HRpeak (*r* = −0.45; *p* = 0.03), while no significant associations were found with CO, CI, or vascular resistance parameters.

## Discussion

In our study population of adult patients after the Fontan procedure, more than one third demonstrated significantly impaired exercise capacity, defined as peak VO_2_ < 16.6 mL/kg/min or <50% of predicted peak VO_2_, which is associated with an unfavorable prognosis (6). Across all analyzed PhysioFlow parameters, we observed a limited adaptive capacity of the Fontan circulation to increasing exercise workload, consistent with findings reported by Legendre et al. (12). Because this was an exploratory observational study, the results support physiological interpretation but do not establish causal pathways.

The mechanisms underlying exercise intolerance are complex and multifactorial; however, a key role is played by the unique hemodynamics of the Fontan circulation, in which, following the palliative creation of a TCPC, cardiac output becomes preload-dependent because pulmonary blood flow relies on passive venous return. Although resting stroke volume and cardiac output may be preserved, chronically reduced preload limits cardiovascular reserve and the ability to augment cardiac output during exercise. Our findings extend previous invasive and imaging studies by providing beat-to-beat characterization of these dynamic responses during incremental exercise in adults (Central Illustration).

**CENTRAL ILLUSTRATION:**
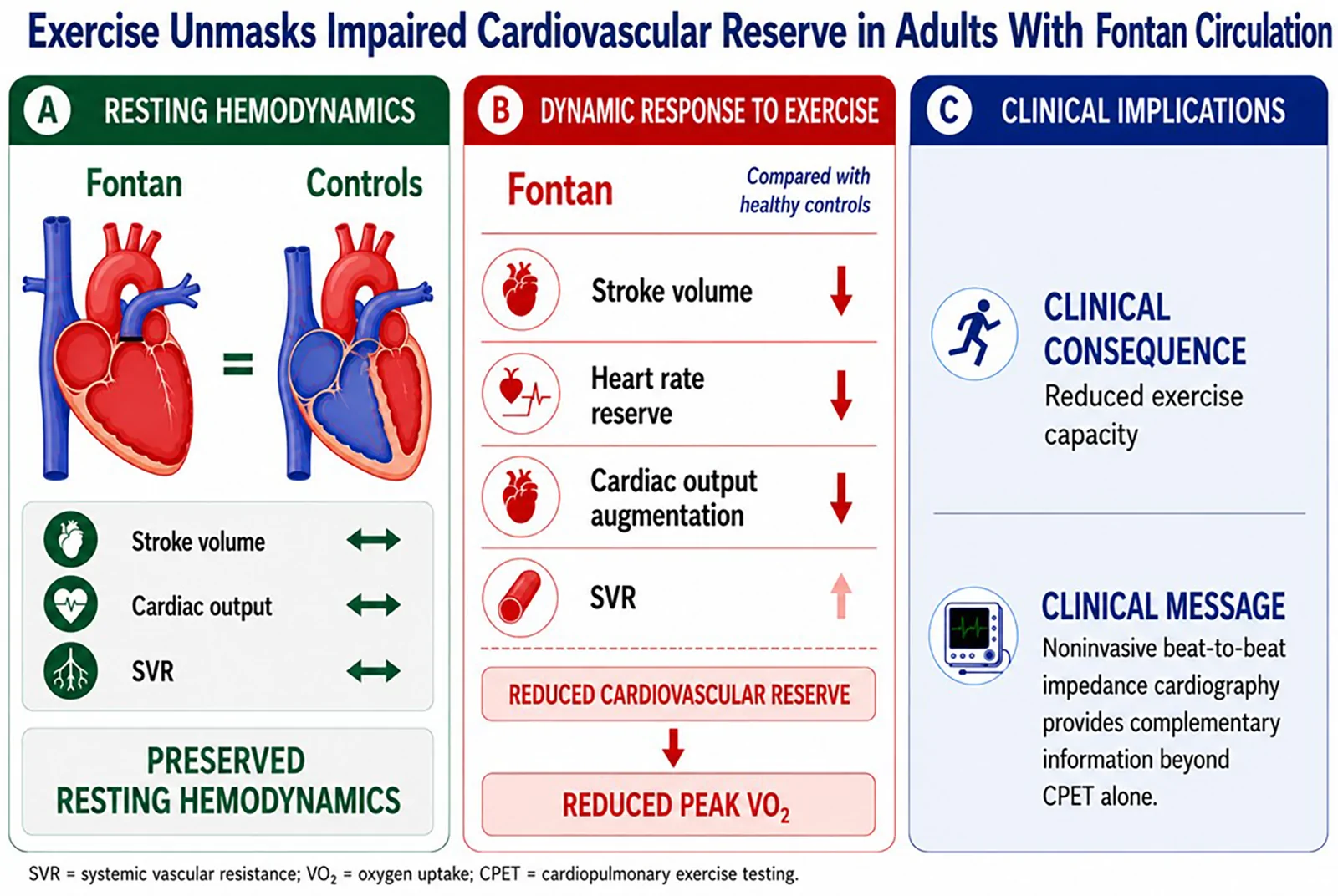
Adults with Fontan circulation exhibit preserved resting central hemodynamics but impaired cardiovascular reserve during exercise. Exercise is characterized by reduced chronotropic response, limited augmentation of cardiac output, and a less pronounced decrease in systemic vascular resistance, resulting in reduced peak oxygen uptake and exercise capacity. These findings highlight the complementary value of noninvasive beat-to-beat impedance cardiography in addition to conventional cardiopulmonary exercise testing. Panel **(A)**, rest; panel **(B)**, exercise response; panel **(C)**, clinical implications.

### Chronotropic response as an important contributor to exercise capacity

Under physiological conditions, increases in cardiac output during exercise are achieved through coordinated augmentation of both stroke volume and heart rate. In the Fontan circulation, however, stroke volume reserve is constrained by the absence of a subpulmonary ventricle and the reliance of pulmonary blood flow on passive venous return. Consequently, heart-rate augmentation becomes closely linked to the ability to increase cardiac output (4, 5). In our study, peak HR and HRR-related variables were associated with exercise capacity and CI, suggesting that chronotropic response is an important marker of reduced exercise reserve. This association should not be interpreted as evidence that impaired sinus node function is the primary cause of exercise intolerance. Claessen et al. demonstrated that lower HR reserve in Fontan patients may reflect abnormal ventricular filling and early stroke-volume limitation, with a chronotropic response that is appropriate relative to metabolic demand (14). Thus, lower peak HR and limited stroke-volume reserve are best understood as interrelated manifestations of the same preload-limited Fontan physiology rather than as independent causal mechanisms (15–17). Furthermore, pharmacotherapy may limit heart rate reserve, as in our study almost 50% of patients with chronotropic incompetence were using beta-blockade.

The interpretation of chronotropic response in Fontan patients should also take into account the marked heterogeneity of this population. Exercise performance is influenced not only by the unique Fontan physiology but also by patient-specific factors, including underlying congenital anatomy, ventricular morphology, Fontan type, fenestration status, pharmacological therapy, and cardiac pacing. Previous studies suggest that patients with a dominant systemic left ventricle generally achieve higher exercise capacity than those with a systemic right ventricle, whereas persistent fenestration may improve preload and cardiac output at the expense of arterial oxygen desaturation (18, 19). Likewise, beta-blocker therapy and cardiac pacing may directly influence chronotropic response during exercise (18). Owing to the relatively small sample size, meaningful subgroup analyses and sensitivity analyses excluding paced patients were not feasible in the present study. Therefore, our findings should be interpreted as reflecting the overall physiological characteristics of a heterogeneous adult Fontan population rather than the effect of individual anatomical or clinical factors. Future studies including larger multicenter cohorts will be required to determine the independent contribution of these variables to exercise hemodynamics.

### Stroke volume as a parameter with limited adaptive reserve

Although stroke volume was significantly reduced at peak exercise in Fontan patients, it did not correlate with CPET-derived parameters. This observation is broadly consistent with Legendre et al. (12), who emphasized heterogeneous stroke-volume profiles during exercise in Fontan patients and reported limited agreement between stroke-volume behavior and conventional CPET indices.

In Fontan physiology, stroke volume often reaches a near-maximal value early during exercise because of limited preload, leaving little capacity for further augmentation (2, 4, 5). In our study, mean SV increased from 78.1 mL at ventilatory threshold to 85.9 mL at peak exercise, representing a modest additional increase of approximately 10%. This pattern suggests limited late stroke-volume reserve rather than a complete plateau.

### Peripheral vascular resistance as a secondary component of hemodynamic regulation

In addition to chronotropic limitation, we observed a less pronounced reduction in systemic vascular resistance during exercise in Fontan patients. While systemic vascular resistance was higher at peak exercise and correlated inversely with exercise capacity, this finding should be interpreted cautiously. Because SVR was calculated from noninvasive blood pressure measurement and impedance-derived CO under a fixed CVP assumption, it may be biased in Fontan physiology, where CVP is elevated and dynamically changes during exercise. In this context, increased systemic vascular resistance is more likely to represent a relative hemodynamic pattern or secondary adaptation to limited cardiac output than a primary determinant of exercise intolerance (20). Reductions in systemic vascular resistance alone are unlikely to substantially increase cardiac output without concomitant improvements in pulmonary blood flow and preload (4, 21).

Increased systemic vascular resistance and venous hypertension represent distinct hemodynamic phenomena, reflecting abnormalities in the arterial and venous circulation, respectively (19).

It should also be acknowledged that chronic disturbances in vascular regulation, including endothelial dysfunction, neurohormonal activation, and microcirculatory alterations, may modulate the vascular response to exercise (22, 23).

### Discrepancy between resting and exercise hemodynamic assessment

A key finding of the present study is the clear dissociation between resting and exercise hemodynamics. Despite largely comparable resting parameters between Fontan patients and controls, substantial abnormalities emerged during exercise (Figure 1).

Our results highlight the limitations of resting assessment in this population and emphasize the importance of dynamic evaluation for detecting clinically relevant impairments. This observation is consistent with previous invasive and imaging studies demonstrating that resting measurements may underestimate the severity of circulatory dysfunction in Fontan physiology (18, 24, 25).

### Consistency of hemodynamic parameters with CPET results

CPET remains the gold standard for the assessment of exercise capacity and an important prognostic tool in patients with Fontan circulation (6, 7, 26). In the present study, a significant association was observed between CPET results and hemodynamic parameters assessed noninvasively. Similar observations were reported by Legendre et al., who highlighted the value of exercise hemodynamic monitoring in revealing heterogeneous circulatory responses in patients with Fontan circulation (12). Takao et al. reported pilot data in 9 Fontan patients and 10 postoperative biventricular controls using impedance cardiography to assess CI during exercise (13). These data support feasibility but should not be interpreted as broad validation of impedance cardiography for adult Fontan exercise hemodynamics. While impedance cardiography does not allow direct assessment of preload or pulmonary hemodynamics, the observed patterns are consistent with and complementary to previously reported invasive and imaging-based studies (13, 27, 28).

### Clinical implications

Our findings suggest that noninvasive, exercise-based hemodynamic assessment may provide valuable insights into the mechanisms underlying exercise intolerance in Fontan patients. Evaluation of chronotropic response, CI, and relative vascular resistance patterns during exercise may help identify patients with impaired cardiovascular reserve who are not recognized by resting assessment alone. However, given the exploratory design, small sample size, absence of outcomes, and lack of invasive validation in this cohort, these observations should not yet be used to guide therapy without confirmation. Potential clinical applications include optimization of pharmacotherapy, individualized exercise prescription, and selection of patients for more advanced hemodynamic evaluation (29).

## Limitations

Several limitations should be acknowledged. First, the sample size was relatively small, reflecting the limited availability of adult Fontan patients undergoing comprehensive exercise testing. Given the exploratory nature of this physiological study, no adjustment for multiple comparisons was performed; therefore, individual associations should be interpreted with appropriate caution and considered hypothesis-generating. Second, hemodynamic measurements were obtained using noninvasive impedance cardiography. Although this method has been validated against reference techniques in selected populations, its accuracy in Fontan physiology during upright treadmill exercise remains incompletely established, and the results should be interpreted primarily in terms of relative changes rather than absolute values. Third, systemic vascular resistance was calculated under the assumption of constant central venous pressure. This assumption may be particularly problematic in Fontan patients, in whom central venous pressure is elevated and dynamically changes during exercise, potentially affecting the accuracy of derived resistance values. Therefore, the observed differences in SVR should be interpreted as reflecting relative hemodynamic patterns rather than direct evidence of abnormal vascular regulation. Fourth, the Fontan cohort was heterogeneous with respect to ventricular morphology, Fontan characteristics, and exercise-induced desaturation. These factors were not systematically evaluated because the limited sample size precluded meaningful subgroup analyses. Finally, the influence of pharmacotherapy, particularly beta-blocker therapy, and cardiac pacing in two patients may have affected chronotropic response and should be considered when interpreting the observed associations.

## Conclusions

Adult patients after the Fontan procedure exhibit reduced hemodynamic reserve that becomes apparent during exercise. Our findings suggest that impaired chronotropic response is closely associated with reduced hemodynamic reserve and lower cardiac index during exercise, but it should be interpreted as part of preload-limited Fontan physiology rather than as an isolated causal mechanism. Altered vascular response may also contribute to the observed exercise pattern, although SVR estimates remain relative because CVP was not directly measured. The limited ability to augment stroke volume appears to represent a characteristic feature of Fontan physiology and may constrain both cardiac output and heart-rate response during exercise. Overall, our findings support a pattern of preserved resting hemodynamics but impaired cardiovascular reserve during exercise in adults with Fontan circulation. Noninvasive exercise hemodynamic assessment may help identify abnormalities not evident at rest and support comprehensive functional evaluation, while larger prospective and invasively validated studies are needed before therapeutic conclusions can be drawn.

## Data Availability

The raw data supporting the conclusions of this article will be made available by the authors, without undue reservation.
